# IN THEIR VOICES: A QUALITATIVE STUDY OF OLDER ADULTS’ PERSPECTIVES OF COVID-19 PANDEMIC

**DOI:** 10.1093/geroni/igac059.2979

**Published:** 2022-12-20

**Authors:** Chih-ling Liou

**Affiliations:** Kent State University, North Canton, Ohio, United States

## Abstract

Older adults are a very heterogeneous group and are likely to experience the pandemic in a variety of ways. This study is to give older adults voices to share their experiences of this pandemic. Data were collected in spring 2022 using semi-structured interviews with 46 adults (74% females) aged 66 and 97 residing in the Midwestern United States. Participants were asked to share how the pandemic affects them, their vision for the future, and how they cope during the pandemic. Participants expressed both positive and negative effects of pandemics. Although they shared more information and examples of the negative effects, most of them did express an optimistic view of the future. The most reported negative effects were few contacts with family and friends, isolation/loneliness, and confinement/restrictions. The most reported positive effects were having more time, getting things done, driving less, and becoming more appreciative. Their perspectives toward the COVID-19 pandemic are varied: some believed that the effects of the pandemic are just temporary, and others compared it to the wars that were much worse. Findings reveal that oldest-old (85+) and old-old (75–84) participants are better at regulating their negative effects compared to the young-old (65–74). Older adults during the pandemic are not just passive help receivers but can be proactive helpers to give advice and coping strategies as well as provide emotional support to others. Older adults’ adaptability during the COVID-19 pandemic should be better understood to reverse the image of their vulnerabilities and promote late-life coping during crises.

